# Supplementary material for: Transparent EuTiO3 films: a possible two-dimensional magneto-optical device
Source: Sci Rep. 2017 Jan 13;7:40621. doi: 10.1038/srep40621 (PMC5234035; doi:10.1038/srep40621)
Supplement: Supplementary Information [file srep40621-s1.doc]

**Transparent EuTiO_3_ films: a possible two-dimensional magneto-optical device**

Annette Bussmann-Holder^1*^, Krystian Roleder^2^, Benjamin Stuhlhofer^1^, Gennady Logvenov^1^, Iwona Lazar^2^, Andrzej Soszyński^2^, Janusz Koperski^2^, Arndt Simon^1^, Jürgen Köhler^1^

^1^Max-Planck-Institut für Festkörperforschung, Heisenbergstr. 1, D-70569 Stuttgart, Germany

^2^Institute of Physics, University of Silesia, ul. Uniwersytecka 4, 40-007 Katowice, Poland

**Supplementary information**

**A Sample preparation and characterization:**

Samples of ETO have been synthesized by repeatedly heating mixtures of Ti_2_O_3_ and Eu_2_O_3_ at 1300°C with intimate grinding in between, which ensures optimal target properties. The films were grown on STO (001) single crystal substrates provided by Crystec Company (Germany). For the PLD ablation process a KrF excimer laser with a wave length of 248 nm was used. The energy density on the target was ~ 1.6 J/cm^2^ and the frequency of the pulsed laser beam was 10 Hz. The deposition rate was ~ 0.257 Å/pulse calibrated by measuring the film thickness with a profilometer. Using a resistive heater the substrate temperature was kept constant at 600°C during the film growth according to the radiation pyrometer reading. While depositing the film an oxygen flow with a flow rate 4.4 sccm was assured, with the pressure in the deposition chamber being 1∙10^-5^ mbar. The STO substrate thickness was 0.1mm and the ETO films were 1000nm thick. The films have been characterized by scanning electron microscopy (SEM) showing a smooth and homogenous surface, atomic force microscopy (AFM) verifying a surface roughness of less than 0.25nm, and x-ray diffraction (Cu Kα_1_ radiation) confirming cubic symmetry at room temperature with no c-axis shrinkage or expansion (Figure SM1). The in-plane resistivities of the films were larger than 10MΩ, and the band gap, as determined by spectroscopic ellipsometry, 4.53±0.07eV [14].


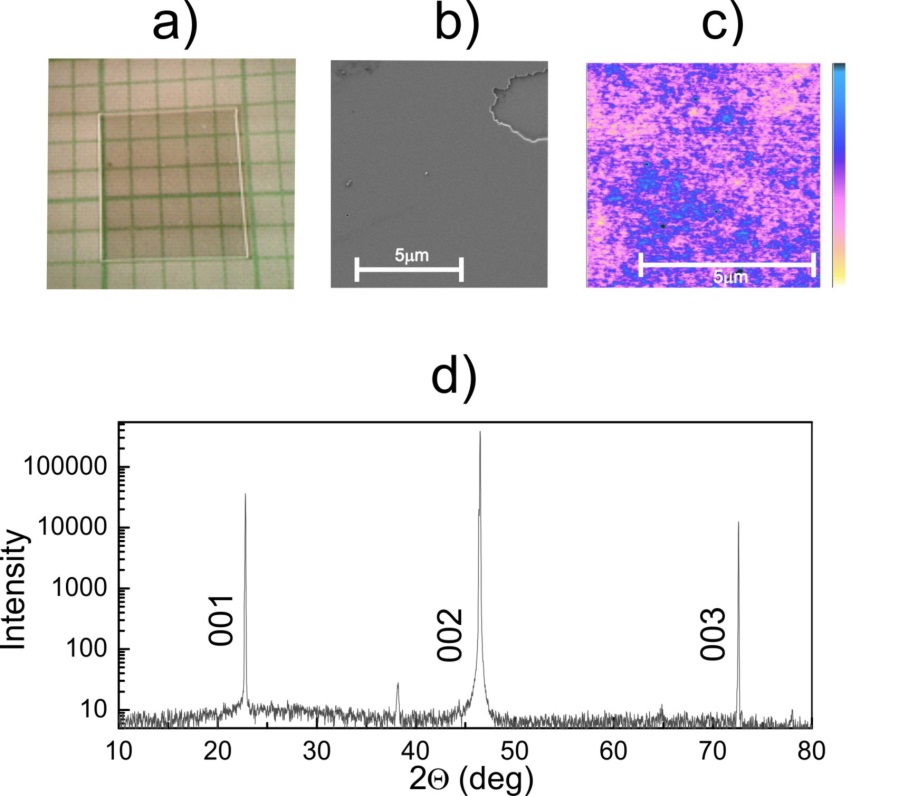


**Figure SM1** a) Typical photo picture of a 0.5 x 0.5 cm^2^ ETO film. b) and c) Scanning electron microcopy SEM picture and atomic force microscopy (AFM) picture of a selected area of the ETO film shown in a), respectively. d) XRD pattern of the ETO film on a STO substrate. The 38.2° Bragg peak stems from the sample holder.

Magnetic susceptibility measurements have been carried out and confirmed the transition to AFM order at T_N_=5.1K.

The cubic – tetragonal transition at T_S_=282K was detected by birefringence Δn measurements (Figure SM2). At T_S_ Δn adopts finite values and increases linearly with decreasing temperature. A deviation of Δn from linearity sets in around T*≈190K and is exemplified in the inset to the figure where the linear part has been subtracted. Another change in slope takes place around 150K, which we tentatively assign to different stacking of domains along the c-direction. The magnetic field effect on the briefringence is shown in Figure SM2b where the huge increase in Δn as compared to Figure SM2a is obvious. In this figure not only the change in slope at T_S_, T* and 160K is evident, but an additional maximum of Δn at T’≈95K is apparent which possibly connects to another symmetry lowering. Since the values of Δn near this maximum are about 2 orders of magnitude larger than Δn of SrTiO­_3_ which undergoes a cubic tetragonal transition at 103.5K (Figure SM2c) this anomaly cannot by connected with any strain from the substrate.

**
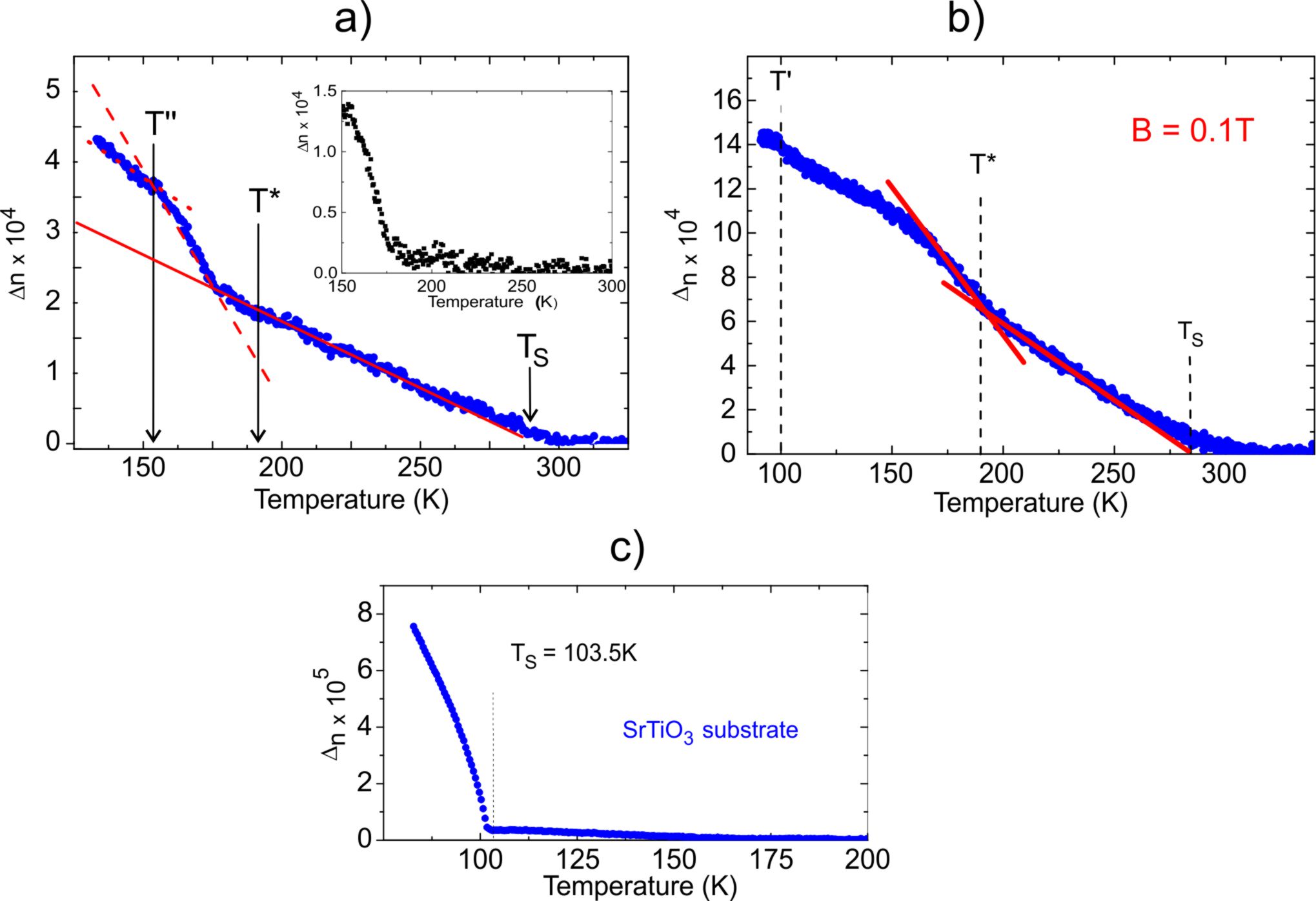
**

**Figure SM2 (a)** Measured birefringence ∆n of the ETO film as a function of temperature. The transition temperatures are marked by vertical lines where T’’ is indicated as well, however, possibly not related to a true phase transition. The straight red line is the behavior of the birefringence as expected from Landau theory. The dashed red line indicates the change in slope below 190K, and the dotted line refers to the next slope changeexpected to stem from different domain stacking along the c-axis. . The inset to **(a)** shows the same as the main figure, however, with the blue data being subtracted by the straight red line. **(b)** Birefringence of a EuTiO_3_ thin film under a magnetic field of 0.1T oriented along the [1-10] direction. T_S_ and T* mark the almost linear dependence Δn(T), whereas T’ is close to a low temperature inflection point of this function which is caused by the diffuseness of the transition points due to the magnetic field. T’’ indicates the crossover temperature between two linear in T Δn regions. **(c)** Measured birefringence of the STO substrate. The dashed vertical line indicates the structural phase transition temperature.

**B The Metripol system and the data analysis**

Birefringence measurements were made on an ETO film with thickness of 1µm oriented in [001] direction and deposited on a single crystal STO substrate. The thickness of the film and substrate together was 85µm. In all measurements we have used a Linkam TMSG600 temperature stage combined with the Metripol Birefringence Imaging System (Oxford Cryosystems). This system consists of a polarizing microscope and a computer controlling number of rotations of the polarizer (usually 10 rotations), an analyzer and a CCD camera measuring light intensity *I* at each position of the polarizer. This intensity is given by the following relation:


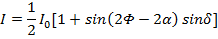


where *I*_o_ is the intensity of light that passes through the sample (transmittance),
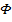
 is the angle of an axis of the optical indicatrix in relation to the pre-determined horizontal axis, and
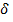
is the phase difference between the polarized light components, and reads:


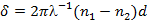
,

where λ is the wavelength of the light and *d* is the thickness of sample (in our case of the thin film). The birefringence is defined as
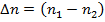
 and was measured as seen in projection down the microscope axis. The interesting feature of the Metripol system is to rectify results from a background stemming from uncorrected optical signals, e.g. originating from glass windows used in TMSG600 stage. Because of this background the final signal is a kind of effective retardation described by [S1]:


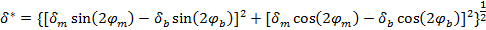


where
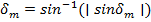
 and
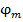
are the phase shift and orientation angle of the sample (here the ETO/STO sample), and
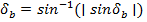
and
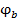
 are the phase shift and orientation angle of the background outside the sample. In this way the birefringence can be detected with a very high sensitivity of the order of 10^-6^.

The main axis of the indicatrix could be oriented 0° or 90° to the [001] direction of the tetragonal unit cell. As a consequence, the measuring light beam passing through the ETO/STO sample was not parallel to the optic axis ([001] direction for the tetragonal symmetry), and the light was split into the ordinary and extraordinary ray. Since an important benefit of this system, in comparison to the conventional crossed polarizer method, is that the orientation of the indicatrix (i.e. the specimen) does not matter and that the absolute value of *Δn* is determined, these two possible orientations of the optic indicatrix are not an obstacle to obtain the temperature dependence of the birefringence. Experimentally, the wavelength of 570nm was used. By measuring several images with varying angle α, it is possible to determine for each pixel position the quantities *I*_0_, sin
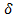
 and φ separately, and then to plot images in false color representing these three values.

The temperature was controlled to within ±0.1K, and the measurements were made with optimal cooling and heating rates not larger than 0.7K per minute. It was confirmed that slower rates did not change the experimental results. Prior to each measurement, the sample was rejuvenated (e.g. to minimize stresses in the sample) at a temperature of 470K for half an hour.

Another benefit of this method is that we can make orientation images which are obtained by subtracting one image from another. In the case of images presented in our paper the subtracted image was taken at 350K, i.e. far above the transition point to the cubic phase. This image was treated as a background produced by the uncorrected optical anisotropy from the optical path or reflections from lenses.

In order to study the effect of a magnetic field on the birefringence, for the above described system a supplement holder has been designed. The sample was placed between two Nd-magnets - with size of 10 (hight) x 3 x 2 mmm or 10 (hight) x 3 x 1.5mm – which were glued to a macor glass ceramic support. The magnetic induction B at the magnets surface was 0.1T and 0.3T, respectively. The B lines were homogenous perpedicular to the height of the magnets. Values of B in the middle of the pair of magnets, i.e. in the position at which regions in size of tens of micrometers were taken for calculations of birefringence, were measured by a Hall sensor. Stronger B fields were obtained by attaching next magnets to both sides of the support.


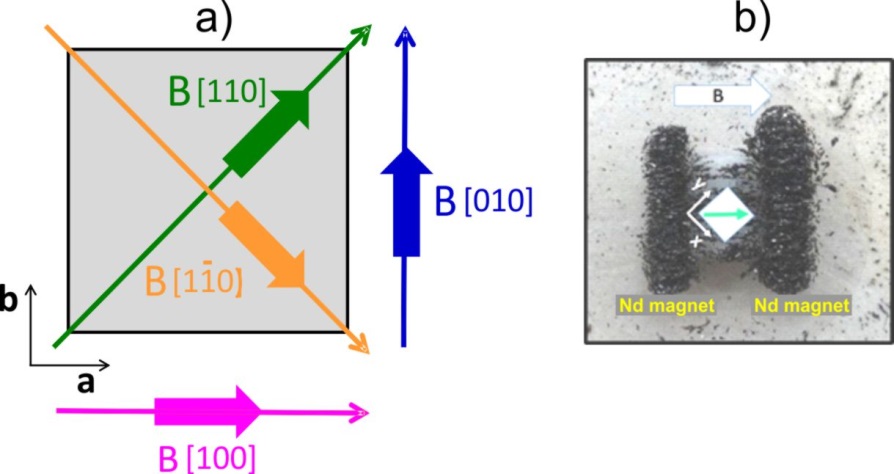


**Figure SM3** **(a)** Geometric configurations of the birefringence Δn measurements in a magnetic field. The picture is a simple visualization (by means of metal fillings) of the B lines perpendicular to the height of the Nd magnets, and **(b)** the orientation of the sample in a magnetic field B along the [110] direction. .

**C Symmetry analysis of the ETO structure**

The symmetry of the low temperature phase of ETO below T* can be derived from the perovskite group-subgroup relations between space groups. A detailed analysis has been carried out H. Bärnighausen and by A. M. Glazer in terms of oxygen octahedral tilting. Using their notations, the probable sequences of phase transitions and of tilts in perovskites is schematically depicted in Figure SM4. Starting from the high temperature cubic structure four continuous pathways to lower symmetries exist together with two first order transitions Since for ETO the transition at T_S_ has been assigned from
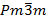
 to
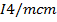
 three continuous lines connect from
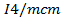
 to subgroups which are either orthorhombic, or tetragonal or monoclinic. The next subgroups are all monoclinic. Since no detailed structural refinements of ETO in a magnetic field exist and synchrotron data are too scarce to detect the symmetry lowering, an exact assignment is at present impossible.


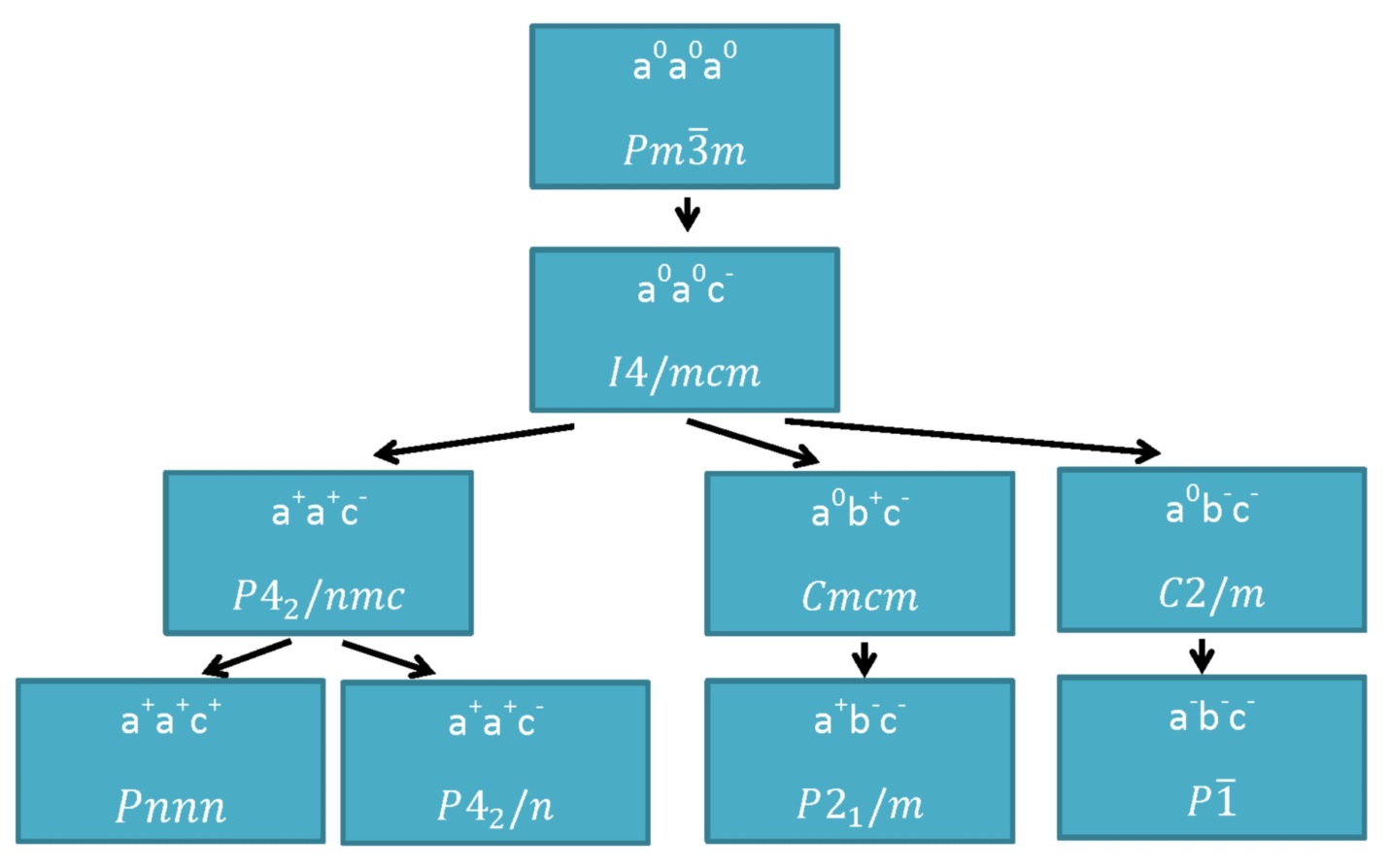


|  |  |  |  |
| --- | --- | --- | --- |

**Figure SM4** Schematic diagram indicating the group-subgroup relationships among the structures formed by the perovskite.

We thus conclude from the birefringence data and the above symmetry considerations that for a magnetic field of H=0.02T the transition at T*=190K is the one from tetragonal to monoclinic symmetry whereas in a field of H=0.1T the transition at T_S_=282K is from the cubic to monoclinic.

**References**

[S1] M. A. Geday and A. M. Glazer, *Birefringence of SrTiO_3_ at the ferroelastic phase transition* J. Phys.: Cond. Mat. 16, 3303-3306 (2004).

[S2] H. Bärnighausen, *Group–subgroup relations between space groups as an ordering principle in crystal chemistry: the ‘family tree’ of perovskite-like structures.* Acta Cryst. A31, part S3, 01.1–9 (1975); H. Bärnighausen, *Group–subgroup relations between space groups: a useful tool in crystal chemistry.* MATCH Commun. Math. Chem. 9, 139–175 (1980).

[S3] A. M. Glazer, *Simple ways of determining perovskite structures* Acta Cryst. A31, 756-762 (1975).
